# Investigation of hypoxia conditions using oxygen-enhanced magnetic resonance imaging measurements in glioma models

**DOI:** 10.18632/oncotarget.16256

**Published:** 2017-03-16

**Authors:** Qi Fan, Cheuk Ying Tang, Di Gu, Jinyu Zhu, Guojun Li, Yingwei Wu, Xiaofeng Tao

**Affiliations:** ^1^ Radiology Department, Shanghai People's Ninth Hospital, Shanghai Jiao Tong University, School of Medicine, Shanghai, China; ^2^ Radiology Department, Mount Sinai School of Medicine, New York, USA; ^3^ Department of Urology, Shanghai First People's Hospital, Shanghai Jiao Tong University, School of Medicine, Shanghai, China; ^4^ Departments of Head and Neck Surgery, University of Texas, M.D. Anderson Cancer Center, Houston, TX, USA

**Keywords:** hypoxia, glioma, oxygen-enhanced magnetic resonance imaging, GLUT-1, pO2

## Abstract

The objective of this study was to determine whether using oxygen-enhanced magnetic resonance imaging (OE-MRI) to assess hypoxia is feasible and whether historical measurements, pO2 changes, and percentage of signal intensity changes (PSIC) are correlated in an animal model of glioma. A total of 25 Sprague-Dawley rats were used to establish C6 brain or subcutaneous glioma model. Nine rats with brain gliomas underwent OE-MRI followed by histopathologic analysis to assess microvessel density and hypoxia. Another 11 rats were underwent OE-MRI and were followed for a survival analysis. Time–T1-weighted MR signal intensity (SI) curves and PSIC maps were derived from the OE-MRI data. High–regions of interests (ROI-h; PSIC > 10%) and low-ROIs (ROI-l; PSIC < 10%) were defined on the PSIC maps. To validate the PSIC map for identifying tumor hypoxia, we subjected an additional 5 rats with subcutaneous glioma to OE-MRI and pO2 measurements. All tumors showed regional heterogeneity on the PSIC maps. For the brain tumors, the time-SI curves for the ROIs-h showed a greater increase in SI than those for the ROIs-l did. The percentage of tumor area with a low PSIC was significantly correlated with the percentage of hypoxia staining and necrosis (r =0.71; P<0.05). ROIs with a higher PSIC typically had more vessels (r=0.88; P<0.05). A significant difference in survival was shown (log-rank P = 0.035). The time-pO2 curves of the subcutaneous tumors were similar to the time-SI curves. PSIC was significantly correlated with pO2 changes (r =0.82; P<0.05). These findings suggest that OE-MRI measurements can be used to assess hypoxia in C6 glioma models. In these models, the PSIC value was correlated with survival, indicating that PSIC could serve as a prognostic marker for glioma.

## INTRODUCTION

The median 5-year survival rate of glioblastoma (GBM) patients has little improved over the past several decades owing to the tumor's heterogeneity and resistance to available treatments [1]. Studies have shown that GBMs can have heterogeneity [2]. Studies with novel glioma animal models have shown that human GBMs have two different invasive, and angiogenic phenotypes. These studies also revealed that high-density tumor cells were in the center of tumor, whereas necrosis and pseudopalisading glioma cells were in the core. Tumor cells were clustered around the neovasculature at the tumor border [2]. Moreover, diffuse tumor cells were able to infiltrate and migrate to the surrounding normal brain parenchyma.

Hypoxia contributes to tumor heterogeneity and induces therapy resistance and poor prognosis [3, 4]. Previous studies have reported that the phenotype of glioma cells can shift from proliferation to invasion and that this shift is probably triggered by hypoxia. In addition, tumor vasculature is highly correlated with hypoxia, and GBMs express high levels of vascular endothelial growth factor (VEGF), especially in areas of necrosis and hypoxia [5]. These findings suggest that hypoxia is critical to the progression of GBM. Tumor cell proliferation leads to necrosis and induces hypoxia. Hypoxia in turn contributes to glioma growth, invasion, and metastasis via multiple pathways, including those that involve glucose transporter 1 (GLUT-1) [6, 7], VEGF, and carbonic anhydrase IX [8], which have been used as biomarkers for cancer. Among these biomarkers, GLUT-1 may be an important independent prognostic indicator [9]. As abnormal angiogenesis is crucial for maintaining an adequate oxygen level and nutrient supply [10], microvascular density is recognized as a specific molecular marker of hypoxia.

Mapping tumor hypoxia could be used to improve therapy, monitor treatment effects, and predict outcomes. Noninvasive imaging studies may be used to help map the regional distribution of hypoxia before surgery or targeted radiotherapy, assess suitable patients for alternative therapies, monitor treatment effects, and predict patient outcomes [4, 5]. However, using noninvasive imaging studies to evaluate hypoxia is challenging. Eppendorf polarographic oxygen microelectrode systems are the gold standard for mapping hypoxia in tumor models, but the invasiveness of these systems limits their clinical use in humans [11–13]. Positron emission tomography (PET) has been used to identify tumor hypoxia in humans but has a low signal-to-noise ratio, and tracers for the method are not commonly available [14, 15].

Magnetic resonance imaging (MRI)–based techniques to identify tumor hypoxia in humans include echo-planar imaging–based, blood oxygenation level–dependent (BOLD) MRI combined with respiratory challenge to change the ratio of oxygenated to deoxygenated hemoglobin [16]. However, the images obtained with this method are severely distorted [17, 18]. Dynamic contrast-enhanced MRI, in which a contrast agent is used to assess perfusion, might be used as an indirect measurement of oxygen saturation [6, 19, 20].

Given the limitations of these methods, there is a great need to develop an easy-to-use, noninvasive imaging method for identifying tumor hypoxia in humans. Oxygen-enhanced MRI (OE-MRI) might be one of the potential techniques since oxygen makes an increase in the longitudinal relaxation rate owing to its paramagnetic properties as well as no significant change in R2* value [21, 22]. In this study, we used a turbo-spin-echo sequence with inversion recovery acquisition (IR-TSE) to obtain OE-MRI data. Then, we quantified the signal changes to measure hypoxia in gliomas. Finally, we investigated the correlations among OE-MRI data, histological measurements, and survival outcomes. In addition, we used the partial pressure of oxygen (pO2) as a reference to validate OE-MRI for hypoxia mapping.

## RESULTS

### Study animals

**Figure 1:**
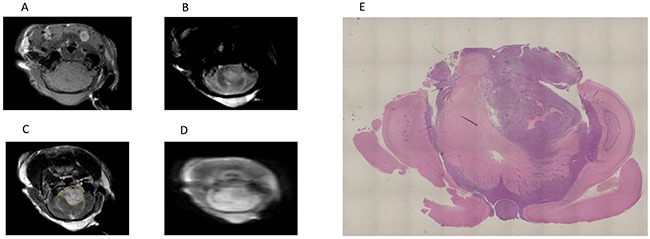
MR images of one representative brain tumor **(A)** T1-weighted MR image. **(B)** T2-weighted MR image. **(C)** Gadolinium-enhanced T1-weighted MR image. The yellow line indicates the tumor. **(D)** IR-TSE image. **(E)** A histological section of a whole brain referred to the MRI plane, where the ROIs were placed.

A total of 25 rats completed the protocol and had paired T1-weighted OE-MRI data. MR images of one representative brain tumor are shown in Figures 1A–1E.

### Regional heterogeneity within brain tumors

**Figure 2:**
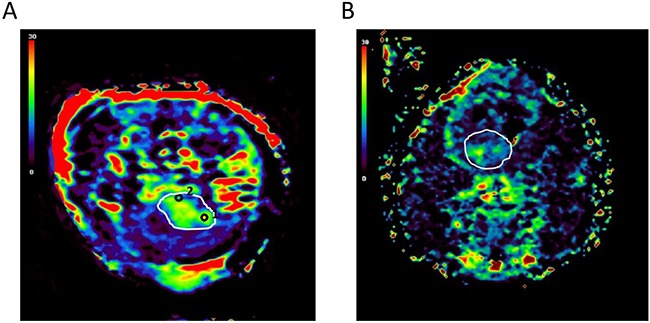
Regional heterogeneity within brain tumors **(A)** PSIC maps obtained for one representative brain tumor show a small proportion of tumor area exhibiting low PSIC (PSIC < 10%). The white line indicates the tumor. Black circles indicate the tumor-ROIs; circle “1” indicates the ROI-h, and circle “2” indicates the ROI-l. **(B)** PSIC maps obtained for one representative brain tumor with a large proportion of area exhibiting low PSIC (PSIC < 10%). The white line indicates the tumor.

Tumor heterogeneity is a critical factor that limits current therapeutic management [23]. All brain tumors showed regional heterogeneity based on responses to oxygen. Regions of interest (ROIs) with a percentage of signal intensity (SI) changes (PSIC) > 10% (high-ROIs, ROIs-h) and ROIs with a PSIC < 10% (low-ROIs, ROIs-l) were determined on the PSIC map. Among all the tumors, proportion of area exhibiting low PSIC (PSIC < 10%) within the tumor was 25%. The PSIC map of one representative brain tumor with ROI placement is shown in Figure 2A. PSIC mapping revealed that all tumors had both ROI-h and ROI-l areas, indicating intratumoral regional heterogeneity and various hypoxia conditions (Figure 2A and 2B).

### Differences of time-SI curves between high-ROIs and low-ROIs

**Figure 3:**
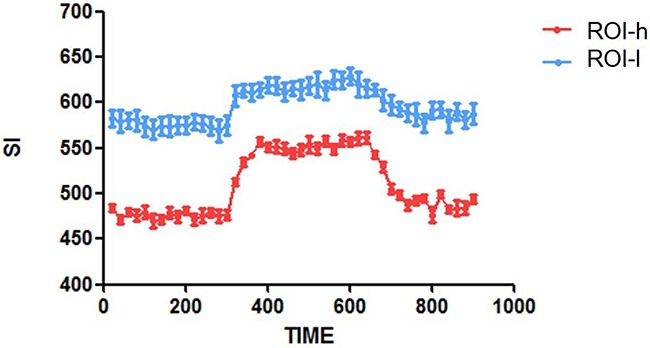
Time-SI curves for the ROIs-h and ROIs-l in one representative brain tumor

Increased T1-weighted MR SI in the tumors during oxygen breathing indicated an increase in the longitudinal relaxation rate. To further investigate differences in SI between ROIs-h and ROIs-l, we calculated time-SI curves for ROIs-h and ROIs-l for a representative brain tumor (Figure 3). Generally, the SIs of both the ROIs-h and ROIs-l increased and then plateaued at a relatively high level during oxygen administration. However, the increase in SI was greater for the ROIs-h than for the ROIs-l. After oxygen administration, the SIs of both the ROIs-h and ROIs-l decreased with time and then stabilized.

### Correlation between PSIC and hypoxia staining

**Figure 4:**
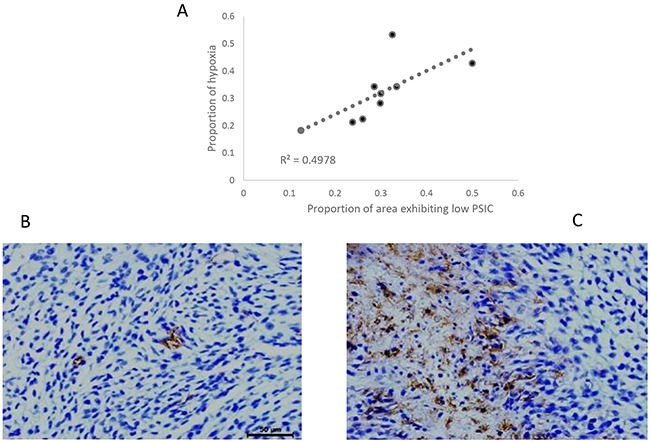
Relationship between PSIC and hypoxia staining **(A)** Correlation between the proportion of area exhibiting low PSIC (PSIC < 10%) and the proportion of area with hypoxia or necrosis (crosses) in gliomas. **(B)** GLUT-1 staining in a ROI-h (magnification ×400). **(C)** GLUT-1 staining in a ROI-l (magnification ×400).

To assess the relationship between PSIC and tumor hypoxia, we subjected whole-brain tissue sections corresponding to MRI slices with marked ROIs (Figure 1E) to histological analysis with staining for GLUT-1, a marker of hypoxia. Tumor areas with low PSIC (PSIC < 10%) were significantly correlated with GLUT-1 staining and necrosis (r =0.71; P<0.05) (Figure 4).

### Correlation between PSIC and historical vascularity measurement

**Figure 5:**
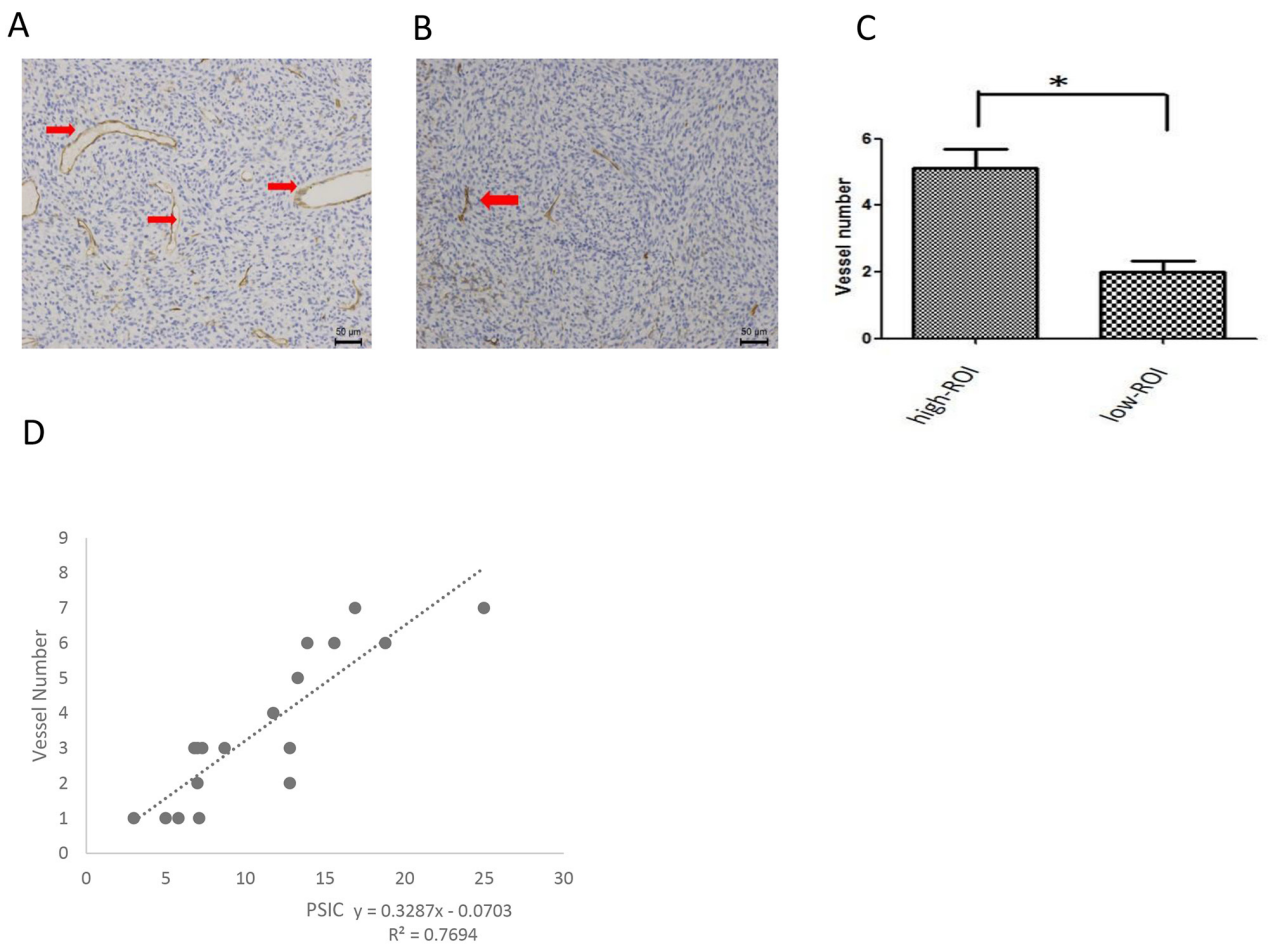
Relationship between PSIC and histological vascularity measurement **(A)** CD34 staining in a ROI-h (magnification ×200). Red arrows indicate vessels. **(B)** CD34 staining in a ROI-l (magnification ×200). Red arrows indicate vessels. **(C)** Mean vessel numbers for ROIs-h and ROIs-l. Error bars represent the standard deviation. **(D)** Correlation between vessel number and PSIC (crosses) in gliomas.

To assess tumor vascularity, we counted the vessels in the ROIs. The mean vessel number of the ROIs-h (5.1 ± 1.7) was significantly higher than that of the ROIs-l (2.0 ± 1.0; P<0.01). Moreover, the PSICs of both the ROIs-h and ROIs-l were significantly correlated with the vessel number (r=0.877; P<0.01) (Figure 5).

### Correlation between survival and PSIC

**Figure 6:**
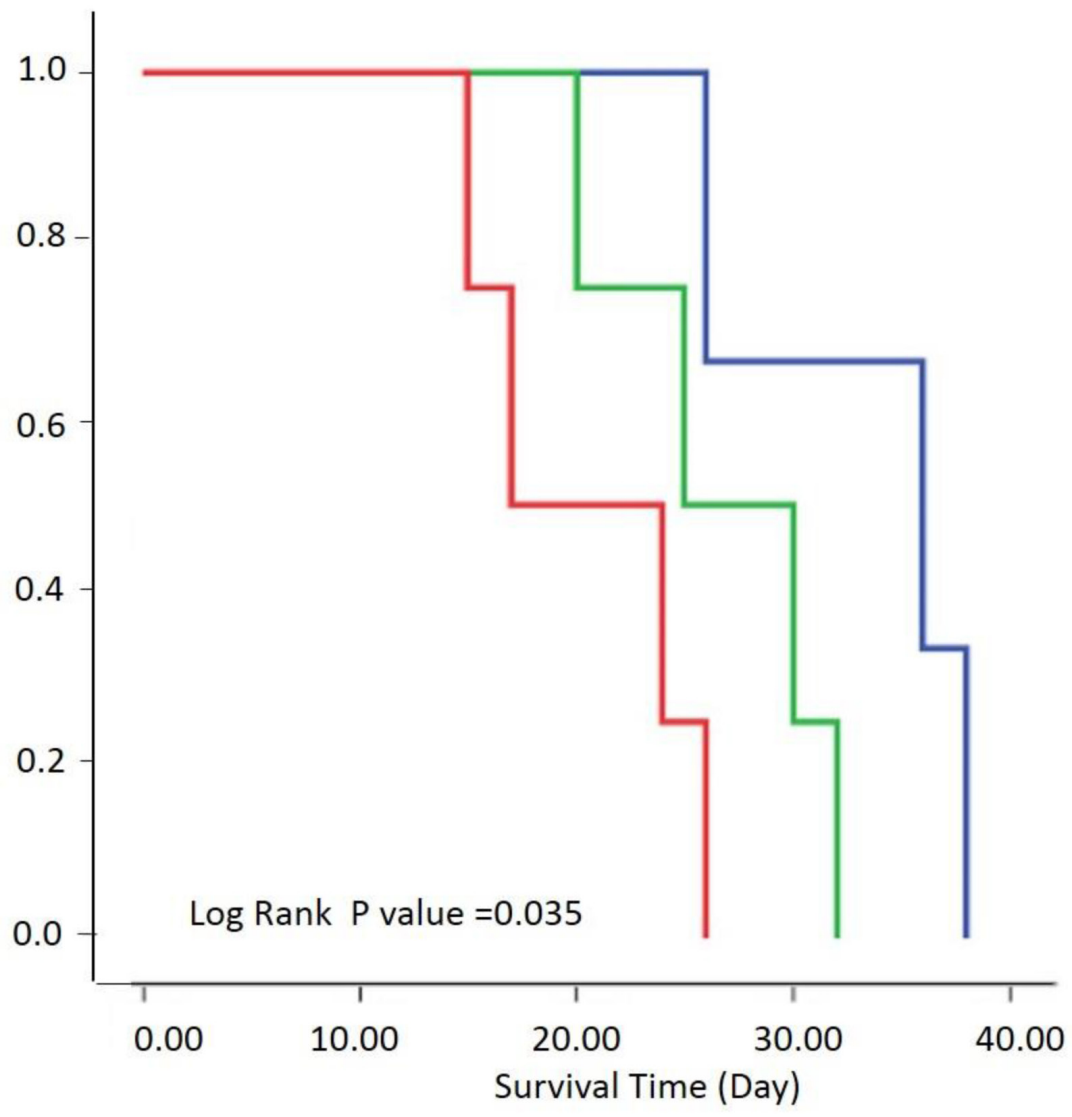
Relationship between survival and PSIC Survival by three groups was performed according to the percentage of area exhibiting low PSIC (PSIC<10%). Kaplan-Meier survival curves of rats with a low percentage (<20%; blue), medium percentage (20–30%; green), or high percentage (>30%; red) of tumor area with low PSIC.

We generated Kaplan-Meier survival curves to assess the correlation between survival and PSIC. The survival curve was performed according to the percentage of area with low PSIC (PSIC<10%). The three subgroups’ median survival times were 36, 25, and 17 days, respectively (Figure 6). The survival rates of the three groups differed significantly (log-rank P=0.035).

### Correlation between PSIC and pO2 measurement in subcutaneous tumors

**Figure 7:**
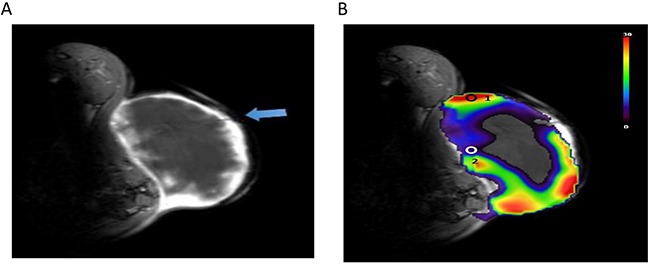
PSIC maps of a representative subcutaneous tumor **(A)** Gadolinium-enhanced T1-weighted MR image. The blue arrow indicates OxyLite probes. **(B)** PSIC maps of a representative subcutaneous tumor. Circles represent ROIs; circle “1” indicates the tumor-ROI, and circle “2” indicates the penumbra-ROI.

To validate the role of PSIC in hypoxia measurement, we used OxyLite probes to measure the change in pO2 (ΔpO2) in the tumors. OxyLite probes were placed in the solid tumor and penumbra areas. The OxyLite probes had slight hypointensity on T1-weighted images (Figure 7A). The PSIC map of one representative subcutaneous tumor with ROI placement is shown in Figure 7B. The OxyLite probe measuring point of penumbra area was mainly located in the hypoxia region (PSIC<10%) delineated by OE-MRI; and measuring point of solid tumor was mainly located in the normoxia region (PSIC>10%) delineated by OE-MRI.

**Figure 8:**
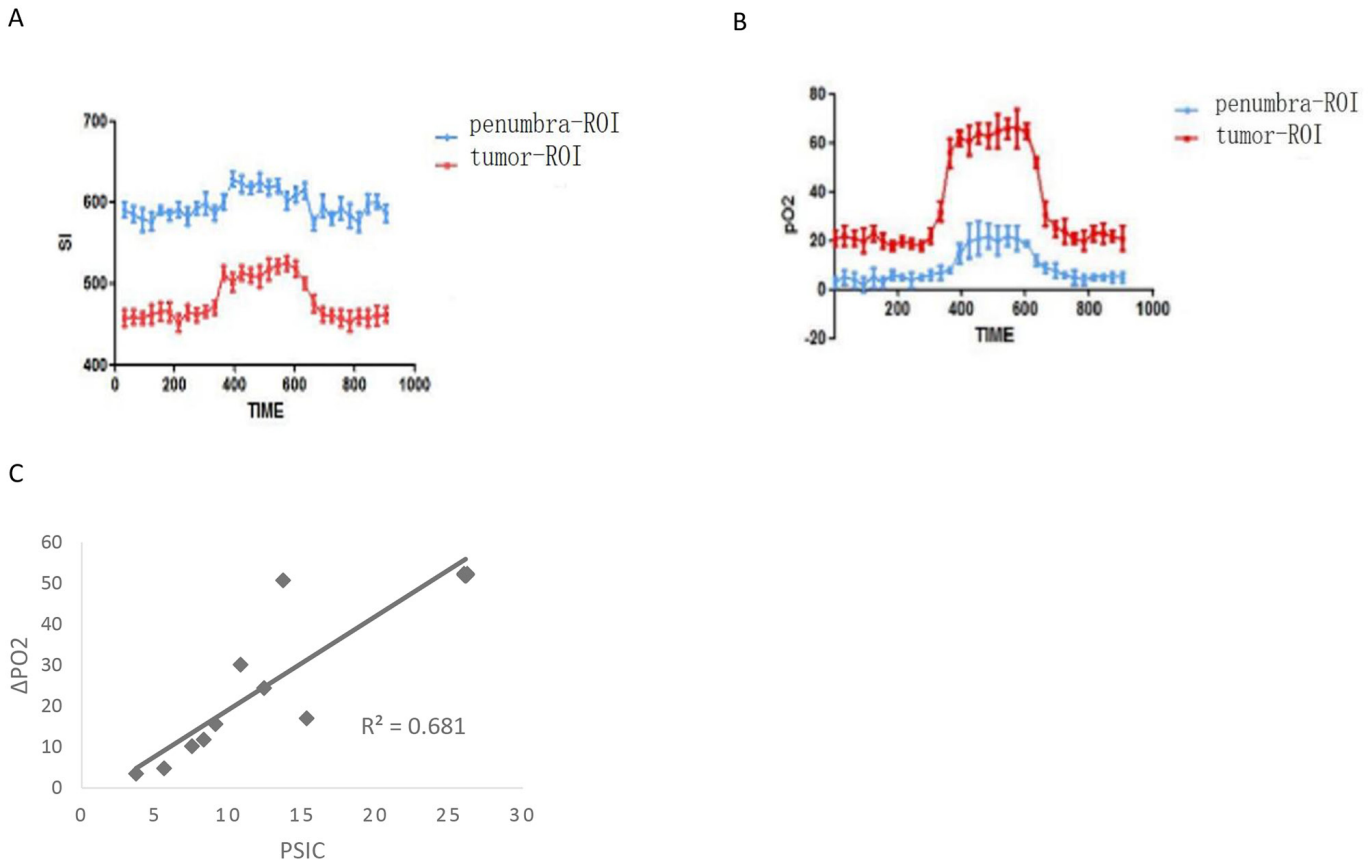
Relationship between PSCI and pO2 measurements in subcutaneous tumors **(A)** Time-SI curves for the tumor-ROI and penumbra-ROI of one representative subcutaneous tumor. **(B)** The pO2-time curves for the tumor-ROI and penumbra-ROI of one representative subcutaneous tumor. **(C)** Correlation between PSC and ΔpO2 (crosses) in subcutaneous tumors.

The pO2s of the solid tumors and penumbra areas were measured over time, and a pO2-time curve was calculated for each area. Similar to the SI-time curve, the pO2-time curve increased during oxygen administration and then gradually decreased and finally plateaued after oxygen administration (Figure 8A and 8B). The mean PSIC of the solid tumors (15.6 ± 5.4) was significantly higher than that of the penumbra areas (6.8 ± 2.0; two-tailed P<0.05). The mean ΔpO2 of the solid tumors (34.8 ± 14.1) was significantly higher than that of the penumbra areas (9.1±4.5; two-tailed P<0.05). The PSIC and ΔpO2 values were significantly correlated (r=0.82; two-tailed P<0.05) (Figure 8C)

## DISCUSSION

Malignant gliomas are characterized by their fast proliferation, infiltration, and invasion into the surrounding brain tissue as well as their abnormal vascularization. Increasing evidence suggests that their heterogeneity is more complex than expected. Many recent studies have reported that glioma cells can change their biological features upon disease recurrence and progression. Many signaling pathways are involved in tumor phenotype shifts among proliferation, invasion, and vascularization phenotypes which lead to heterogeneity. Hypoxia is an important tumor microenvironment factor that affects tumor invasiveness. Hypoxia may enhance tumor cells’ capacity to seek growth conditions suitable to induce tumor angiogenesis or tumor cells’ migration into normal parenchyma for oxygen. Therefore, hypoxia has been shown to be associated with a higher likelihood of metastasis and recurrence [24]; increased resistance to chemotherapy and radiation therapy [25]; and worse patient survival [26, 27]. Therefore, there is an urgent need for noninvasive hypoxia measurement in humans. The current gold standard for hypoxia assessment is measurement with probes such as Eppendorf oxygen microelectrodes [28] or OxyLite probes [29]; however, this method is invasive and is not appropriate for use in humans.

The ideal method for hypoxia assessment is imaging. Several methods have been evaluated for noninvasive detection of hypoxia. These methods include PET using specific tracers and MRI techniques such as BOLD MRI, diffusion MRI, etc. 18F-fluoromisonidazole (18F-MISO) was widely used as PET imaging agent for hypoxia imaging [14, 15]. Limited availability of the tracers and relatively slow clearance from normoxic tissues makes them unsuitable for routine clinical use. In comparison, MRI has a higher popularity, better spatial resolution, and less cost than PET. BOLD-MRI indirectly measures oxygenation changes in tissues, which is based upon measurement of the MR transverse relaxation rate (R2*) of water in blood and surrounding tissues [16]. This method may suffer from distortion, to the extent of which depends on organ site, the control of visceral motion, and precise protocol adopted [17, 18]. OE-MRI is potentially superior to BOLD-MRI [30]. Hyperoxic gases can be vasoactive except the limitations of BOLD-MRI aforementioned. The vasoactive effect may cause perfusion hematocrit changes, which may independently affect the BOLD signal [31]. Diffusion-weighted MRI measures the mobility of water within tissues at a cellular level. Recently, the value of HIF-1α has been reported to have a significantly positive correlation with the value of cellular density, while a significantly negative correlation with the value of apparent diffusion coefficient (ADC) in 34 patients with cerebral astrocytoma [32]. However, the relationship between hypoxia in gliomas and diffusion-weighted imaging measurements is warranted for further investigation.

In this study, we used a noninvasive quantitative, OE-MRI–based method to identify normoxic and hypoxic regions in tumor and investigated the correlation between signal changes and histological examination. We also validated OE-MRI for hypoxia mapping by directly measuring pO2.

Oxygen inhalation has two opposite effects on tissue *R1*. First, a high level of oxygen, especially 100% oxygen, dissolved in blood plasma and interstitial fluid increases the tissue *R1*. This increase is considered to be linearly dependent on the concentration of dissolved oxygen [33]. The weak longitudinal relativity of deoxygenated hemoglobin has been observed [34]. Second, in contrast, increased oxygenated hemoglobin compartmentalized in red blood cells decreases the tissue *R1* to some extent [35]. For ROIs-h of brain tumor or tumor-ROIs of subcutaneous tumors, the decrease in the tissue *R1* caused by oxygenated hemoglobin is very small compared with that caused by molecular oxygen. For ROIs-l of brain tumors or penumbra-ROIs of subcutaneous tumors, these two opposite effects occur together within an imaging voxel, and the reduction in the paramagnetic effect of deoxygenated hemoglobin molecules might not be ignored. Linnik et al used OE-MRI to identify regional tumor hypoxia, and they found that tumor regions with decreasing *R1* were positively correlated with areas of hypoxia [36]. In this study, we identified the hypoxic area with less increasing signal, which was confirmed by direct quantifiable measurements of pO2. In contrast, we found that tumor regions with increasing *R1* were correlated with areas of hypoxia, which we confirmed by directly measuring pO2. The difference between the findings of Linnik et al and those of the present study may be attributable to the higher flow rate of oxygen used in the present study [36].

We also demonstrated that the OE-MRI data were correlated with some of the immunohistochemistry biomarkers and survival. Four key observations in the present study provide evidence that the PSIC map can help identify tumor hypoxia: 1) the proportion of tumor with a low PSIC (PSIC<10%) was strongly correlated with GLUT-1 expression and necrosis; 2) high PSIC was significantly correlated with high vessel density; 3) the survival times of rats grouped according to the percentage of tumor area with low PSIC differed significantly; and 4) PSIC was significantly correlated with pO2 changes in subcutaneous tumors. Hypoxic staining was observed distant from the vessels, where tissue oxygenation is insufficient. Hypoxia helps upregulate the biomarkers essential for angiogenesis and may be essential to the promotion of both pathological and physiological vessel formation [37].

Other studies in some metastatic and advanced cancers of the pelvis and abdomen have reported results similar to those of the present study [38]. Animal models of different tumors exhibited SI changes after undergoing T1-weighted OE-MRI [39, 40], which can also serve as a non-invasive imaging method in normoxic tissue, including arterial blood plasma and cerebrospinal fluid [21, 41]. In such studies, molecular oxygen worked as an exogenous contrast agent to identify hypoxic tumor regions.

The tumor microenvironment influences tumor growth and metastasis and therapy effectiveness [42]. In glioma patients, the tumor environment is complicated owing to the hypoxia in different areas. Preoperative MRI routinely identifies a necrotic center, a presumed hypoxic penumbra, an active tumor area, and peritumoral edema in gliomas. The extent of necrosis and vascular endothelial proliferation in gliomas is useful for predicting outcomes in patients [43]. Hypoxic tissues in gliomas are thought to play a part in the disease's resistance to radiation therapy and chemotherapy. Routine gadolinium-enhanced MRI is used to identify the anatomical features and grade of a glioma. The extent of peritumoral edema and necrosis on such imaging can be used to predict clinical outcomes in patients with newly diagnosed malignant glioma [44]. However, routine MRI alone is insufficient to acutely predict histological grades or tumor types [45]. Dynamic contrast-enhanced MRI provides valuable information about blood flow, tumor vascularity, and permeability. Previous studies have suggested that capillary heterogeneity in areas of active tumor and interstitial volume in areas of peritumoral edema can help identify gliomas with increased hypoxia and proliferation preoperatively [6].

Our study had some potential limitations. First, the rat models used for histological examinations had orthotopic tumors, whereas the models used for pO2 measurement had subcutaneous tumors, and the oxygen dynamics of brain tumors may differ from those of peripheral tumors. Second, the numbers of rats used in the study, especially in the experiments measuring pO2, were small.

In conclusion, the findings of this study provide evidence that OE-MRI can produce reliable measurements to detect tumor hypoxia in a rat model. Further work is warranted to determine whether OE-MRI can be used clinically to identify brain tumor hypoxia in humans.

## MATERIALS AND METHODS

This study was approved by our institution's Institutional Animal Care and Use Committee. A total of 25 male Sprague-Dawley rats (190~220 g; SLAC Laboratories, Shanghai, China) were used to establish C6 brain or subcutaneous glioma model in total.

### Cell culture and brain tumor implantation

Rat C6 GBM cells were obtained from the Cell Bank of the Chinese Academy of Science (Shanghai, China) and grown in culture consisting of Dulbecco's modified Eagle's medium (DMEM; Sigma, USA) with 10% fetal bovine serum (Gibco, America) and 1% penicillin-streptomycin (Sigma). Cultures were maintained in a 37°C humidified atmosphere containing 5% CO2. Cells were routinely sub-cultured twice a week until confluence.

For tumor implantation, the cells were trypsinized by exposure to 0.25% trypsin–ethylenediaminetetraacetic acid and suspended in DMEM without additives. Twenty rats were anesthetized with 10% hydrated chlorine aldehyde and placed in a stereotaxic device. For each rat, a hole was drilled 1 mm lateral and 2 mm anterior to the bregma. About 1 × 10^6^ cells in 10 μL of PBS were injected into the cortex 6 mm below the dura at a rate of 2 μL/min.

### MRI protocol

All 20 rats underwent MRI 2 weeks after tumor implantation. Each rat was anesthetized and underwent femoral vein cannulation for contrast agent administration, and then placed head-first in the supine position into a 3.0-Tesla MRI scanner (Ingenia, Philips Healthcare, Best, The Netherlands) with a 4-channal animal coil, with the center of the head aligned with the center of the radiofrequency coil. Body temperature was maintained at 37.0°C with a heating pad and monitored closely. Pulse and oxygen saturation were monitored closely and stabilized as necessary. Normal room air was administered through a non-rebreather mask fit firmly over the mouth and nose. First, low-spatial-resolution multi-section MRI was performed to confirm the location of the brain. Then, axial T2-weighted TSE MRI (TE/TR, 100/1700 ms; field of view [FOV], 80×80 mm; matrix, 192×96; four 2-mm-thick axial slices) was used for tumor localization. A dynamic OE-MRI series with an IR-TSE sequence (TE/TR, 48/9575 ms; inversion time, 1915 ms; FOV, 80×80 mm; matrix, 192×96; half-Fourier, 6/8; one 2-mm-thick axial slice) was acquired over three stages: 1) a 5-min pre-oxygen stage, during which the animals breathed normal air (21% oxygen); 2) a 5-min 100% oxygen stage, when the animals breathed 100% oxygen; and 3) a 5-min post-oxygen stage, when animals again breathed normal room air. Axial T1-weighted TSE MRI (TE/TR, 23/500 ms; FOV, 80×80 mm; matrix, 192×96; four 2-mm-thick axial slices) was also performed before and after the administration of 0.2 mmol/kg gadopentetate dimeglumine.

### Image analysis

PSIC maps were generated pixel-by-pixel using software developed in-house with the MATLAB platform (MathWorks, Natick, MA). PSICs were calculated using the equation

$$\text{PSIC}=\left(\text{SI }{\text{O}}_{2,\;\max}-\text{SI }{\text{O}}_{2,\text{ pre}}\right)/\text{SI }{\text{o}}_{2,\text{pre}}\times 100\%$$ (1)

where and SI O_2, pre_ is the mean SI value of all time points during the pre-oxygen stage and SI O_2, max_ is the SI at the peak of the SI curve during the 100% oxygen stage for each voxel. Tumors were manually delineated on the PSIC map referred to T2-weighted images and T1-weighted images following gadolinium administration. ROIs with volumes of 0.98 mm^3^ were chosen randomly and defined as either ROIs-h (PSIC > 10%) or ROIs-l (PSIC < 10%) (Figure 1B). The proportion of tumor area exhibiting PSIC < 10% was calculated.

### Survival analysis and histological analysis

Of the 20 rats that underwent MRI, 11 rats were closely followed for the survival analysis. Survival analysis was performed by Kaplan-Meier method and evaluated using the log-rank test. The remaining 9 rats with neurologic signs were humanely killed for tumor histology and immunohistochemistry analyses. These rats were first placed under deep isoflurane anesthesia; their brains were then removed, fixed with 4% paraformaldehyde overnight, and embedded in paraffin. Vessels in tumor were visualized by staining the histological sections with an antibody against mouse CD34 (1:150; Abcam), an endothelial cell marker, and counted. Hypoxic areas in tumor were visualized by staining the histological sections with an antibody against rabbit GLUT-1 (1:100; Abcam), a marker of hypoxia, using the Vectastain ABC kit (Vector Laboratories). The histological sections and the MRI planes were compared with the PSIC maps, and ROIs were selected. The vessels in the ROIs-h and ROIs-l were counted manually. Areas that stained positive for GLUT-1 were considered hypoxic. The proportions of necrosis and hypoxia within whole tumor sections were calculated. A light microscope (Nikon Instruments, Melville, NY) was used to photograph the stained sections at 200× magnification. A trainable segmentation plugin of the Fiji software program) [46] or the ImageJ software program (http://imagej.nih.gov/ij/) was used to analyze CD34 staining and GLUT-1 staining.

### Subcutaneous tumor cell injection and ROI selection

To validate OE-MRI for hypoxia mapping, we used pO2 as a standard. Owing to the difficulty of measuring pO2 in brain tumors, we established subcutaneous glioma models. C6 GBM cells (approximately 1 × 10^6^ in 1 mL of DMEM) were injected subcutaneously into the flanks of 5 male Sprague-Dawley rats. Two weeks after tumor cell injection, T2- and T1-weighted images were obtained with the same protocols used to assess the brain tumors, described above. Four areas of the subcutaneous tumors were identified: 1) the necrotic center (the non-enhancing tumor center); 2) the presumed hypoxic penumbra (the enhancing area around the necrotic center); 3) the active tumor zone (the enhancing area at the outer margin of the tumor); and 4) peritumoral edema (the non-enhancing area that surrounds the active tumor zone and has a bright appearance on T2-weighted images). ROIs were selected in the active tumor and presumed hypoxic penumbra and defined as tumor-ROIs and penumbra-ROIs, respectively.

### Dynamic oxygen enhancement measurements and pO2 measurements for subcutaneous tumors

PO2 measurements and OE-MRI were performed simultaneously. To measure pO2, we used the OxyLite™ Pro system (Oxford Optronix, Oxford, UK), which detects fluorescence with a fiber-optic sensor and measures the fluorescence lifetime, which is inversely proportional to the dissolved oxygen concentration and is interpreted to provide an absolute value for pO2. The OxyLite probe, a 23G surgical steel-encased sensor that was calibrated by the manufacturer prior to delivery, was placed in tumor-ROIs and penumbra-ROIs. A nose mask was used to administer normal room air or 100% oxygen. OE-MRI was acquired over 1) a 5-min pre-oxygen stage, 2) a 5-min 100% oxygen stage, and 3) a 5-min post-oxygen stage as described above. ΔpO_2_ values were calculated using the equation

$$\Delta {\text{pO}}_{2}={\text{pO}}_{2,\text{max}}-{\text{pO}}_{2,\text{pre}}$$ (2)

where pO_2, pre_ is the mean pO_2_ value of all time points during the pre-oxygen stage and pO_2, max_ is the mean pO_2_ value of all time points during the 100% oxygen stage for each voxel. PSIC maps were derived from the IR-TSE images. The PSICs of tumor-ROIs and penumbra-ROIs were calculated.

### Statistical analysis

A paired Student t-test was performed to compare vessel numbers and ΔpO_2_ values between different ROIs. The Spearman rank correlation coefficient was calculated to determine correlations between OE-MRI variables and histological counts and ΔpO_2_ values. For 2-sided tests, *P* values <0.05 were considered statistically significant. All statistical analyses were performed using the MedCalc software package (version 14.8.1, Mariakerke, Belgium).

## Abbreviations

OE-MRI: oxygen-enhanced magnetic resonance imaging
PSIC: percentage of signal intensity changes
GLUT-1: glucose transporter 1;SI, signal intensity
ROI: region of interest
GBM: glioblastoma
VEGF: vascular endothelial growth factor
IR-TSE: turbo-spin-echo sequence with inversion recovery acquisition
DMEM: Dulbecco's modified Eagle's Medium (DMEM)
